# Maternal Folic Acid Supplementation and the Risk of Congenital Heart Defects in Offspring: A Meta-Analysis of Epidemiological Observational Studies

**DOI:** 10.1038/srep08506

**Published:** 2015-02-17

**Authors:** Yu Feng, Song Wang, Runsen Chen, Xing Tong, Zeyu Wu, Xuming Mo

**Affiliations:** 1 Department of Cardiothoracic Surgery, The Affiliated Children's Hospital of Nanjing Medical University, Nanjing, Jiangsu, China; 2 Atherosclerosis Research Center, Key Laboratory of Cardiovascular Disease and Molecular Intervention, Nanjing Medical University, Nanjing, Jiangsu, China

## Abstract

Epidemiological studies have reported conflicting results regarding the association between maternal folic acid supplementation and the risk of congenital heart defects (CHDs). However, a meta-analysis of the association between maternal folic acid supplementation and CHDs in offspring has not been conducted. We searched the MEDLINE and EMBASE databases for articles cataloged between their inceptions and October 10, 2014 and identified relevant published studies that assessed the association between maternal folate supplementation and the risk of CHDs. Study-specific relative risk estimates were pooled using random-effects or fixed-effects models. Out of the 1,606 articles found in our initial literature searches, a total of 1 randomized controlled trial, 1 cohort study, and 16 case-control studies were included in our final meta-analysis. The overall results of this meta-analysis provide evidence that maternal folate supplementation is associated with a significantly decreased risk of CHDs (RR = 0.72, 95% CI: 0.63–0.82). Statistically significant heterogeneity was detected (*Q* = 82.48, *P* < 0.001, *I*^*2*^ = 79.4%). We conducted stratified and meta-regression analyses to identify the origin of the heterogeneity among the studies, and a Galbraith plot was generated to graphically assess the sources of heterogeneity. This meta-analysis provides a robust estimate of the positive association between maternal folate supplementation and a decreased risk of CHDs.

Congenital heart defects (CHDs) are the most common congenital malformations, affecting nearly 1% of live births worldwide^1^. CHDs represent approximately one-third of all congenital anomalies and are the leading cause of perinatal mortality^2^. Although tremendous breakthroughs in cardiovascular diagnostics and cardiothoracic surgery have been achieved over the past century, leading to increased survival for newborns with CHDs, the etiology of most congenital heart defects remains unknown.

Several chromosomal anomalies, certain maternal illnesses, and prenatal exposures to specific therapeutic drugs are recognized risk factors. It is difficult to establish the role of a single factor because the cause of a defect is believed to be multifactorial in many cases; for example, some cases may result from a combination of environmental teratogens with genetic and chromosomal abnormalities^3^. A review published in 2007 provided a summary of the current literature on noninherited risk factors for CHDs^4^. CHDs comprise several distinct subtypes (e.g., conotruncal defects, artioventricular septal defect, and septal defects), and there is a potential for etiologic heterogeneity. Thus, it is not surprising that studies that have examined individual categories of CHDs have come to different or even opposite conclusions.

More than a decade ago, the preventive effects of maternal folate supplementation on the occurrence and recurrence of neural tube defects was documented in several studies^5^,^6^. Primarily because the benefit of folic acid supplementation in preventing neural tube defects in women of childbearing age was shown to be conclusive, folic acid fortification of flour and grain products began in 1998^7^. Maternal multivitamin supplements containing folic acid reduce the risk of neural tube defects, and evidence suggests that maternal folic acid supplementation may also be associated with benefits for other reproductive outcomes, including the incidence of CHDs. Recently, there has been a steep increase in the number of maternal folic acid supplementation studies with the occurrence of CHDs as the primary health outcome; several studies have demonstrated positive associations, whereas others have not.

An increasing number of studies to date have focused on the association between maternal folic acid supplementation and the incidence of CHDs; however, the results have been ambiguous, perhaps due to inadequate sample sizes. Thus, we conducted a meta-analysis to quantitatively assess the effect of maternal folic acid supplementation on the risk of CHDs.

## Results

### Study characteristics

**Figure 1:**
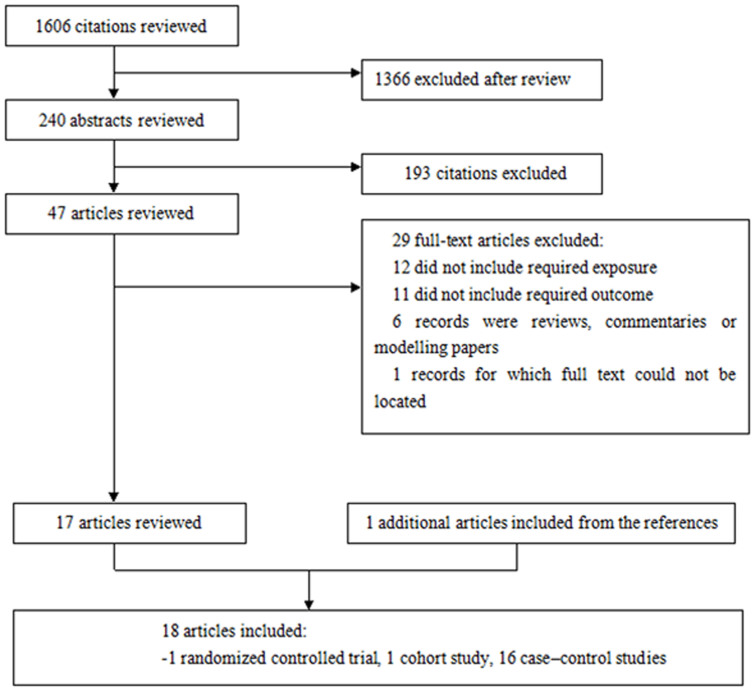
Study selection procedures for a meta-analysis of maternal folate supplementation and the risk of congenital heart defects (CHDs) in offspring.

Our literature search strategy generated 1,606 citations. Of these, 18 were used in the final analysis, representing 18,500 incident cases (Figure 1). All of the studies were published between 1995 and 2013. These studies included 1 randomized controlled trial^8^, 1 cohort study^9^, and 16 case-control studies^10^,^11^,^12^,^13^,^14^,^15^,^16^,^17^,^18^,^19^,^20^,^21^,^22^,^23^,^24^,^25^. The main characteristics of the included studies are presented in Table 1. As shown in Table 1, 9 studies were conducted in the United States, 8 in Europe, and 1 in China. In the 16 case-control studies, the number of cases investigated varied from 77 to 3,278, and the number of control subjects ranged from 250 to 38,151.

### Maternal folate supplementation and CHDs

**Figure 2:**
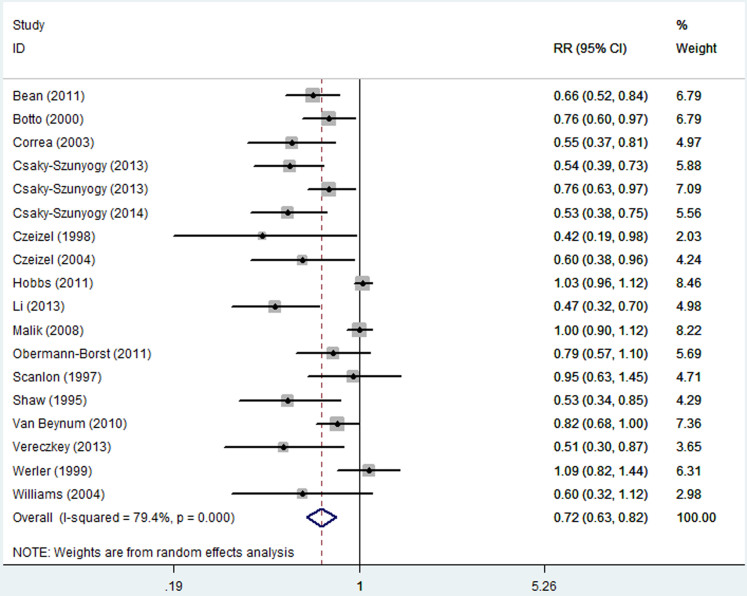
Relative risk (RR) estimates for the association between maternal folate supplementation and the risk of CHDs. Meta-analysis random-effects estimates were used. The sizes of the squares reflect the weighting of the included studies. Bars represent 95% confidence intervals (CIs). The center of the diamond indicates the summary effect; the left and right points of the diamond indicate the 95% confidence interval.

**Figure 3:**
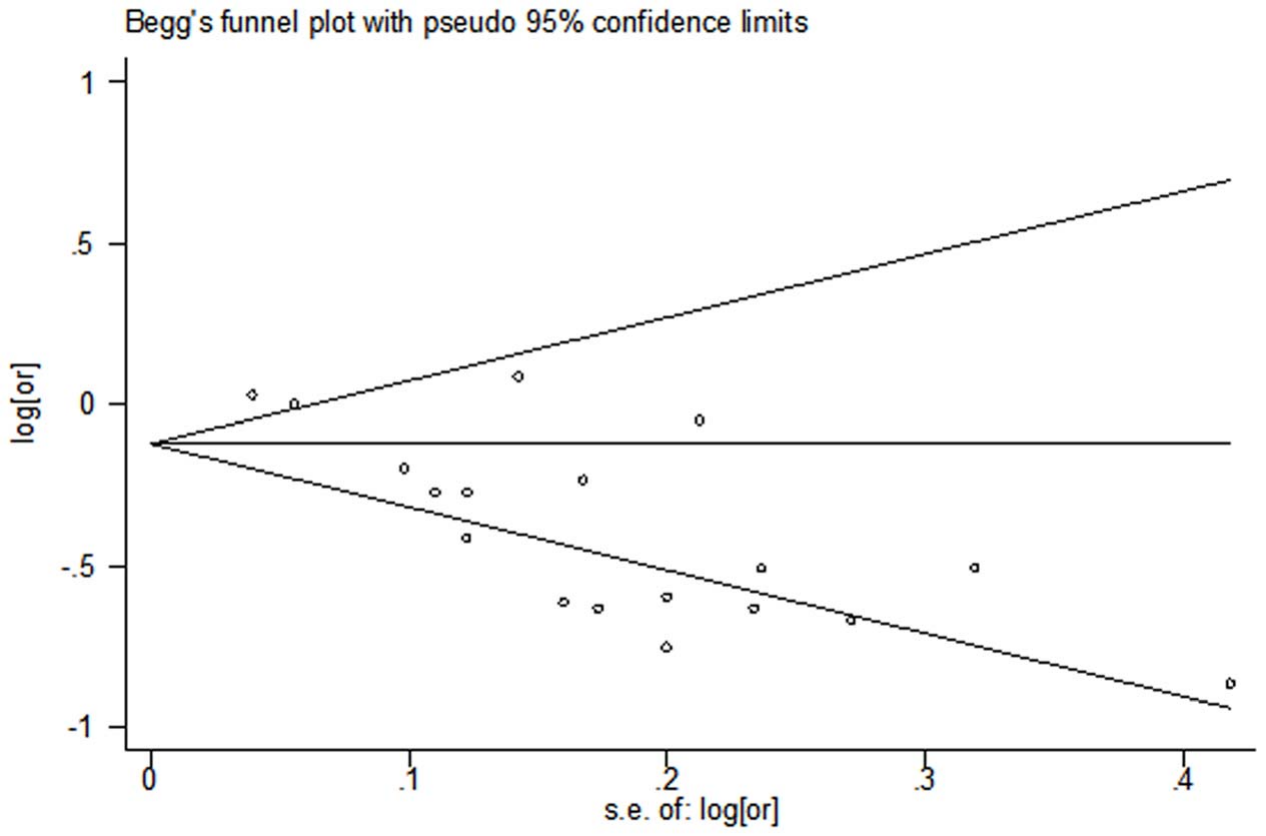
Begg's test of studies examining the association between maternal folate supplementation and the risk of CHDs.

**Figure 4:**
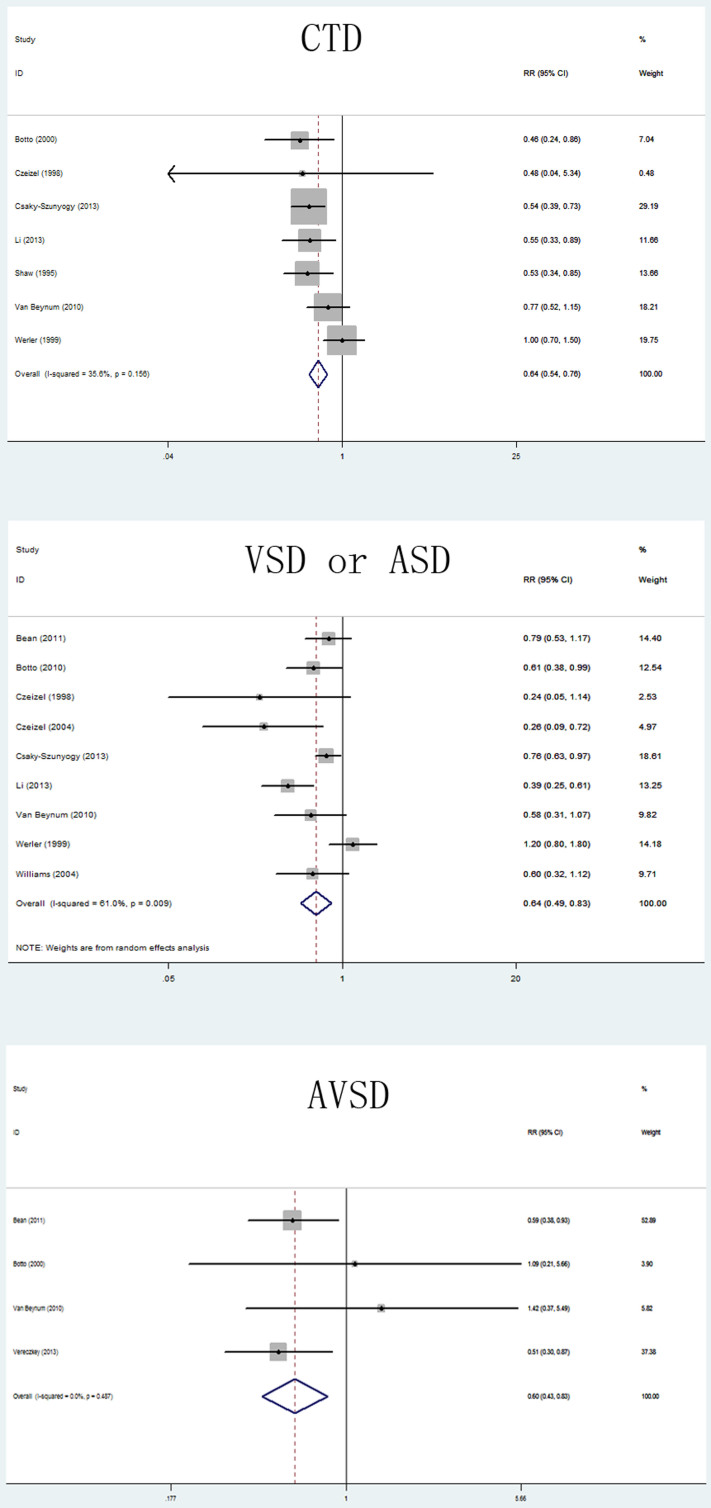
Relative risk (RR) estimates for the association between maternal folate supplementation and the risk of individual subtypes of CHDs (CTD; ASD or VSD; and AVSD). Meta-analysis random-effects estimates were used. The sizes of the squares reflect the weighting of the included studies. Bars represent 95% confidence intervals (CIs). The center of the diamond indicates the summary effect; the left and right points of the diamond indicate the 95% confidence interval.

The overall results of this meta-analysis provided evidence for a significant decrease in the risk of CHDs with maternal folate supplementation (RR = 0.72, 95% CI: 0.63–0.82; Figure 2). Statistically significant heterogeneity was detected (*Q* = 82.48, *P* < 0.001, *I*^*2*^ = 79.4%), with no publication bias (Begg's test: *P* = 0.198; Figure 3). The 18 study-specific relative risks ranged from a low of 0.69 (95% CI: 0.59–0.80, *Q* = 76.40, *P* = 0.000, *I*^*2*^ = 79.1%), after omission of the study by Malik et al.^18^, to a high of 0.74 (95% CI: 0.65–0.84, *Q* = 72.29, *P* = 0.000, *I*^*2*^ = 77.9%), after omission of the study by Li et al.^17^. In stratified analyses, the corresponding pooled RRs were not materially altered in any stratification (Figure 4, Table 2).

### Heterogeneity Analysis

To clarify the sources of heterogeneity, we conducted a sensitivity analysis. However, *I*^*2*^ did not decrease substantially when any individual study was removed. Subsequently, a meta-regression was performed using a Knapp-Hartung modification, and we found that differences in geographical region may contribute to the heterogeneity we observed (*P* = 0.025). We further created a Galbraith plot to graphically assess the sources of heterogeneity (Supplementary Figure S1). A total of 8 studies were identified as the primary sources of heterogeneity. Once the outlying studies were excluded, the heterogeneity was effectively removed (*I*^*2*^ = 31.9%); however, the corresponding pooled RRs were not materially altered (RR = 0.78, 95% CI: 0.69–0.89).

## Discussion

To our knowledge, this is the first quantitative meta-analysis to evaluate the association between maternal folate supplementation and the risk of CHDs. Overall, the findings of our meta-analysis suggest that maternal folate supplementation is significantly associated with a decreased risk of CHDs (RR = 0.72, 95% CI: 0.63–0.82). Moreover, these results were consistent across most of the subgroup analyses (Table 2).

Although the specific biological mechanisms underlying the relationship between maternal folate supplementation and the risk of CHDs remain to be determined, some relevant evidence has been published to date. It has been hypothesized that impaired folate and/or homocysteine metabolism interferes with the development of the heart, possibly by affecting neural crest cells. Methylenetetrahydrofolate reductase (MTHFR), which is a critical folate-metabolizing enzyme, plays an important role in processing amino acids. A C→T substitution is commonly found at position 677 in the MTHFR enzyme and results in a substitution of valine for alanine; this substitution causes impaired folate binding and reduced activity of the MTHFR enzyme^25^. The effect of the MTHFR 677TT genotype on homocysteine levels is more pronounced with low folate status^26^. In 1999, Kapusta et al.^27^ were the first to link maternal hyperhomocysteinemia to an increased risk for CHDs. More recently, Hobbs et al. studied mothers whose children were born with CHDs and identified homocysteine, S-adenosylhomocysteine, and methionine levels as the most important biomarkers predictive of the presence of CHDs^28^. Hernandez-Diaz et al.^29^ showed that periconceptional intake of medications acting as folic acid antagonists, including anti-epileptic agents and dihydrofolate reductase inhibitors, doubled the risk of cardiovascular defects. In vitro studies found that impaired folate and homocysteine metabolism affects neural crest cell formation and migration^30^,^31^. Tang et al.^32^ demonstrated that impaired folic acid transport results in extensive apoptotic cell death in the developing heart; apoptotic cells were shown to be concentrated in the truncus arteriosus and interventricular septum and were thus anatomically restricted to the two regions in which most congenital heart defects are found. Folic acid may play a role in the migration of the cardiac neural crest cells that contribute to the formation of the truncus arteriosus and its division into the pulmonary artery and aorta and thus likely affects the generation of conotruncal defects in particular^33^,^34^. The precise effects of folate supplementation on cardiac morphogenesis are unclear, so it is important to corroborate this hypothesis with evidence from clinical and population-based studies.

Although the potential role of folic acid in the prevention of neural tube defects was reported as early as 1980, public health campaigns have resulted in preconception supplementation in only one-third of pregnant women^35^, partly because one-half of all pregnancies are unplanned. Presently, widely publicized recommendations by various authorities suggest that women should supplement their diet with daily doses of at least 0.4 mg of folate (4 mg for women at higher risk) to reduce the risk of delivering a child with neural tube defects (NTDs)^36^. In many centers, women are advised to begin taking prenatal vitamin supplements when they decide to attempt to conceive. The optimal dose of periconceptional folic acid supplements to prevent CHD cannot be deduced from our study or previous studies because most women had taken supplements containing at least 0.4 mg. Whether a lower or higher dose would be more effective is difficult to explore because 0.4 mg is the level that has been advised for preventing NTDs. There is growing evidence that because mothers are becoming heavier (i.e., because maternal BMIs are increasing), the recommended daily dose of folate will need to be increased to maintain a similar preventive effect^37^.

Some limitations of our study must be taken into account. First, 1 randomized controlled trial, 1 cohort study, and 16 case-control studies were included in our meta-analysis, and we extracted our raw data primarily from several case-control studies, which were susceptible to selection and information biases. In addition, our meta-analysis was limited to studies published in English, so our results may have been affected by a lack of data from studies published in other languages. Thus, our conclusions must be considered carefully. Second, although no evidence of publication bias was found, heterogeneity was among the studies included in the analysis, and this heterogeneity may affect the interpretation of the overall results. In this study, we conducted sensitivity analyses to explore the sources of this heterogeneity by removing one study at a time from our pooled analysis. However, heterogeneity could not be fully removed by the exclusion of any individual study. Moreover, study results may vary with geographical regions, publication periods, sample sizes, CHD subtypes, and other risk factors. Thus, we performed meta-regression and subgroup analyses to further investigate the sources of the heterogeneity that we observed. We found that the heterogeneity in the study results could partly be attributed to the geographical region in which the studies that we examined were conducted. We also created a Galbraith plot to assess the heterogeneity that we observed and to identify potential outlying studies. A total of 8 studies were identified to be the primary contributors to the heterogeneity in the analysis. After excluding these outlying studies, the heterogeneity was effectively removed, whereas the corresponding pooled RRs were not materially altered, indicating that the overall results regarding maternal folate supplementation were statistically stable.

Our study has several important strengths. First, to our knowledge, this is the first meta-analysis to report an association between maternal folate supplementation and the risk of CHDs. Moreover, our literature search was conducted using multiple databases, and the references from the retrieved articles were carefully examined to find any additional studies that may have been of interest. Thus, our study included data for 18,500 cases, which is enough to have sufficient statistical power to investigate the potential association between maternal folate supplementation and the risk of CHDs. Another strength of our study is that although heterogeneity exists in our meta-analysis, we conducted a number of sensitivity, subgroup, and Galbraith plot analyses and found that our results were stable.

In summary, this study provides evidence that maternal folate supplementation is positively associated with a decreased risk of CHDs. However, more prospective studies, particularly in developing countries, are needed to further investigate the association between maternal folate supplementation and the risk of CHDs, especially with regard to the different subtypes of CHDs.

## Methods

### Literature search

A computerized literature search was conducted by two independent investigators (Feng and Tong) using the MEDLINE and EMBASE databases to find articles catalogs from the inceptions of these databases through October 10, 2014. We searched for relevant studies using the following search strategy: (“Multivitamins” OR “Vitamin” OR “Folate” OR “Folic Acid”) AND (“abnormalities” OR “birth defects” OR “congenital anomaly” OR “malformations” OR “congenital malformations” OR “congenital heart defect” OR “Heart Abnormality” OR “Malformation of heart” or “CHD”) AND (“maternal” OR “mother” OR “periconceptional” OR “pregnant” OR “gestation”). In addition, we searched for studies that investigated a broad range of environmental teratogens and CHDs and examined the relevant references and review articles that were found. We were thus able to identify relevant information found in other related studies. We followed published quality standards for conducting and reporting meta-analyses^38^.

### Eligibility Criteria

We selected articles that (1) were original epidemiologic studies (i.e., case–control, cohort or RCT), (2) examined the association between periconceptional folic acid use and either CHDs overall or any one of the CHD subtypes in infants, (3) were published in the English language, (4) reported RRs (i.e., risk ratios or odds ratios) and associated 95% confidence intervals (CIs) or provided raw data from which these measures could be calculated, and (5) defined CHDs or one of the CHD subtypes as an outcome. Articles that reported results from more than one population were considered to consist of separate studies, with 1 study for each population investigated. When multiple articles were found to examine the same study, we included in our study the article with the most applicable information and the largest number of cases. We excluded non-peer-reviewed articles, experimental animal studies, ecological assessments, correlation studies, and mechanistic studies.

### Data extraction

Data extraction was conducted separately by two reviewers (Feng and Wang) working independently. If differences of opinion arose, these were resolved by discussion between the two reviewers. The studies that met the inclusion criteria were reviewed to retrieve relevant information. Relevant information included author names, the year of publication, the geographic region in which the study was conducted, the period in which data were collected, the study design, the sample size, case classification information, exposure and outcome assessments, adjusted estimates and their corresponding 95% CIs, and confounding factors that were controlled for by matching cases or adjustments in the data analysis. When no adjusted estimates were available, we extracted a crude estimate. If no estimate of relative risk was provided in a given study, we recalculated odds ratios or risk ratios and 95% CIs from the raw data presented in the study using standard equations.

To assess study quality, we used a 9-star system based on the Newcastle-Ottawa Scale^39^. Our system judges a study based on three broad characteristics: the selection of study groups, the comparability of study groups and the ascertainment of the exposure or outcome of interest for case-control and cohort studies, respectively. We defined a high quality study as one with a quality score greater than or equal to 7.

### Statistical analysis

We used study-specific relative risks as summary statistics for the association between maternal folate supplementation and CHD risk. To simplify the procedure, an RR was used to represent all reported study-specific results from cohort studies, and an OR was used to represent results from case-control studies. Cochran's *Q* and *I*^*2*^ statistics were used to test for heterogeneity among studies^40^. If there was evidence of heterogeneity (*P* < 0.05 or *I*^*2*^≧56%), a random-effects model was used, which provided a more appropriate summary estimate for heterogeneous study-specific estimates. If the study revealed no evidence of heterogeneity, a fixed-effects analysis was conducted, and an inverse variance weighting was applied to calculate summary RR estimates^41^.

We conducted subgroup analyses based on study design (i.e., RCT or cohort versus case-control studies), geographical region (i.e., USA, Europe, and China), number of cases (i.e., ≤1,000 versus >1,000), publication period (i.e., before 2010 versus 2010 or after), primary focus of the study (i.e., whether the title or abstract refers to folate supplementation as the focus of the study, yes versus no), and study quality (i.e., low versus high quality). We evaluated heterogeneity between subgroups by meta-regression analysis. A *P*-value less than 0.05 for the meta-regression analysis was considered to indicate a significant difference between subgroups. Finally, we conducted sensitivity analyses to explore whether a specific study strongly influenced the results, by excluding one study at a time.

Publication bias was assessed via visual inspection of a funnel plot for asymmetry using both Egger's linear regression^42^ and Begg's rank correlation^43^ methods. For both tests, significant statistical publication bias was defined to be indicated by a *P*-value of <0.05. All statistical analyses were performed using STATA software (version 11.0; StataCorp, College Station, Texas, USA).

## Author Contributions

X.M.M. conceived and designed the experiments. Y.F., S.W. and X.M.M. performed the experiments. Y.F., S.W., R.S.C., X.T. and Z.Y.W. analyzed the data. R.S.C. and Z.Y.W. contributed software, hardware and analysis tools. Y.F. and S.W. wrote the paper. X.M.M. will provide access to the full-text article.

## Supplementary Material

Supplementary Information Supplementary Figure S1

## Figures and Tables

**Table 1:** Summary risk estimates for the association between maternal folate supplementation and the risk of CHDs in offspring

First author, year	Region	Study design	No. of cases/controls^a^	Period	OR/RR	95% CI	Types of CHDs	Adjustment variables^b^
Bean, 2011	USA	CC(P)	566/552	Before pregnancy or within the first 4 weeks of pregnancy	0.59	0.38–0.93	AVSD	Race, sex, alcohol use, smoking
					0.59	0.39–0.90	ASD	
					0.79	0.53–1.17	VSD	
Botto, 2000	USA	CC(P)	958/3029	3 months before through3 months after conception	0.76	0.60–0.97	CHDs	Infant's period of birth, race, chronic diseases
					0.46	0.24–0.86	CTD	
					1.09	0.21–5.66	AVSD	
					0.59	0.38–0.94	Septal	
					0.50	0.14–1.79	ASD	
					0.61	0.38–0.99	VSD	
Correa, 2003	USA	CC(P)	3278/3029	3 months before through3 months after conception	0.55	0.37–0.81	CHDs	Period of birth, maternal age, race, smoking, alcohol use
Czeizel, 1998	Hungary	RCT	2471/2391	3 months before pregnancy	0.42	0.19–0.98	CHD	No
					0.48	0.04–5.34	CTD	
					0.24	0.05–1.14	VSD	
Czeizel, 2004	Hungary	Cohort	3056/3056	1 month before through3 months after conception	0.60	0.38–0.96	CHDs	Parity, chronic diseases, history of previous unsuccessful pregnancies
					0.26	0.09–0.72	VSD	
Csaky-Szunyogy, 2013	Hungary	CC(P)	598/902	First trimester	0.54	0.39–0.73	CTD	Age, parity, maternal employment status
Csaky-Szunyogy, 2013	Hungary	CC(P)	1661/2534	First trimester	0.76	0.63–0.97	VSD	Age, parity, maternal employment status
Csaky-Szunyogy, 2013	Hungary	CC(P)	302/469	First trimester	0.53	0.38–0.75	LOVT	Age, parity, maternal employment status
Hobbs, 2011	USA	CC(P)	417/250	During pregnancy	1.03	0.96–1.12	CHDs	BMI, age, smoking, alcohol
Li, 2013	China	CC(H)	358/422	3 months before through2 months after conception	0.47	0.32–0.70	CHDs	Residence, age, education, family history, planned pregnancy
					0.39	0.25–0.61	VSD	
					0.55	0.33–0.89	CTD	
Malik, 2008	USA	CC(P)	3067/3947	1 month before through2 months after conception	1.00	0.90–1.12	CHDs	Residence
Obermann-Borst, 2011	Netherlands	CC(P)	282/308	4 weeks before conception to 8 weeks thereafter	0.79	0.57–1.10	CHDs	No
Scanlon, 1997	USA	CC(P)	126/679	During pregnancy	0.95	0.63–1.45	OTD	Parity, marital status, drug use
Shaw, 1995	USA	CC(P)	207/481	1 month before to 2^nd^ month of gestation	0.53	0.34–0.85	CTD	Race, age, education, gravidity, alcohol use, cigarette smoking
Van Beynum, 2010	Netherlands	CC(P)	611/2401	4 weeks before conception to 8 weeks after conception	0.82	0.68–1.00	CHDs	Age, BMI, education, smoking, alcohol use
					0.62	0.47–0.82	Septal	No
					0.58	0.31–1.07	VSD	
					0.54	0.31–0.94	ASD	
					0.77	0.52–1.15	CTD	
					1.42	0.37–5.49	AVSD	
Vereczkey, 2013	Hungary	CC(P)	77/38151	First trimester	0.51	0.30–0.87	AVSD	Age, parity
Werler, 1999	USA	CC(H)	343/521	1 month before through1 month after conception	1.00	0.70–1.50	CTD	Age, education, race, planned pregnancy, nausea and vomiting during pregnancy, geographic center
					1.20	0.80–1.80	VSD	
Williams,2004	USA	CC(P)	122/3029	3 months before through3 months after conception	0.60	0.32–1.12	VSD	No

CC: case-control study; CHDs: congenital heart defects; VSD: ventricular septal defect; ASD: atrial septal defect; TOF: tetralogy of Fallot; TGA: D-transposition of the great arteries; HLHS: hypoplastic left heart syndrome; COA: coarctation of the aorta; AVSD: atrioventricular septal defect.

^a^Reported number of cases and control subjects with available exposure information.

^b^Adjustment variables: A, maternal age; B, maternal race/ethnicity; C, marital status; D, maternal education; E, parity; F, smoking; G, coffee consumption; H, infant's year/month of birth; I, intake of multivitamins; J, stress; K, folic acid intake/dietary folate; L, infant gender; M, maternal body mass index; N, family history of congenital heart defects; O, maternal residence; P, maternal occupation; Q, insurance; R, cases and controls matched by birth hospital/geographic region, birth month/age, race, or sex.

**Table 2:** Characteristics of studies examining the association between maternal folic acid supplementation and the risk of CHDs in offspring

Subgroup analysis	No. of studies	Summary RR (95% CIs)	*P*^1^	*I*^2^(%)	*P*^2^
Summary pooled estimate	18	0.72 (0.63–0.82)	<0.001	79.4	
Design					0.284
Case-control	16	0.73 (0.64–0.84)	<0.001	80.4	
Cohort or RCT	2	0.55 (0.37–0.82)	0.458	0.0	
Geographic region					0.025
USA	9	0.83 (0.72–0.96)	<0.001	77.9	
Europe	8	0.70 (0.63–0.78)	0.096	42.3	
China	1	0.47 (0.32–0.70)	-	-	
Publication period					0.545
Before 2010	9	0.75 (0.61–0.92)	0.001	69.7	
2010 or after	9	0.68 (0.55–0.84)	<0.001	85.7	
Number of cases					0.830
≤500	9	0.71 (0.56–0.91)	<0.001	81.6	
>500	9	0.71 (0.60–0.84)	<0.001	74.8	
Primary focus					0.405
Yes	11	0.80 (0.70–0.93)	<0.001	75.9	
No	7	0.74 (0.60–0.90)	<0.001	85.1	
Quality assessment					0.115
High quality studies (scores ≥7)	11	0.79 (0.69–0.90)	0.002	64.3	
Low quality studies (scores <7)	7	0.59 (0.41–0.85)	<0.001	88.6	
CHD subtypes					
CTD	7	0.64(0.54–0.76)	0.156	35.6	
VSD or ASD	9	0.64(0.49–0.83)	0.009	61.0	
AVSD	4	0.60(0.44–0.83)	0.487	0.0	
Confounding Factors					
Age	10	0.68(0.55–0.84)	<0.001	85.6	
Parity	7	0.67(0.56–0.79)	0.039	54.8	
Race	5	0.71(0.56–0.91)	0.013	68.2	
Maternal alcohol	5	0.73(0.56–0.95)	<0.001	86.1	
Maternal smoking	5	0.73(0.56–0.95)	<0.001	86.1	

^1^*P*-value for heterogeneity within each subgroup.

^2^*P*-value for heterogeneity between subgroups in a meta-regression analysis.

Abbreviations: RR: relative risk; CI: confidence interval.
