# Supplementary material for: Maternal Folic Acid Supplementation and the Risk of Congenital Heart Defects in Offspring: A Meta-Analysis of Epidemiological Observational Studies
Source: Sci Rep. 2015 Feb 17;5:8506. doi: 10.1038/srep08506 (PMC4330542; doi:10.1038/srep08506)

Maternal Folic Acid Supplementation and the Risk of Congenital Heart Defects in Offspring: A Meta-Analysis of Epidemiological Observational Studies

Yu Feng, Song Wang, Runsen Chen, Xing Tong, Zeyu Wu and Xuming Mo

Supplementary Figure S1. Galbraith plots for maternal folate supplementation and CHD risk.


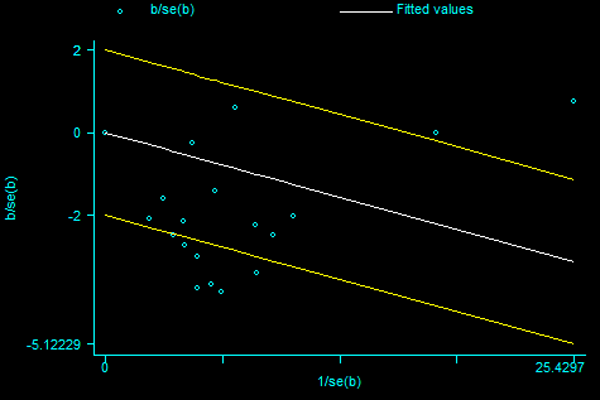

Supplement: Supplementary Information — Supplementary Figure S1 [file srep08506-s1.docx]
